# Adherence, Safety, and Effectiveness of Medical Cannabis and Epidemiological Characteristics of the Patient Population: A Prospective Study

**DOI:** 10.3389/fmed.2022.827849

**Published:** 2022-02-09

**Authors:** Lihi Bar-Lev Schleider, Raphael Mechoulam, Inbal Sikorin, Timna Naftali, Victor Novack

**Affiliations:** ^1^Clinical Research Center, Soroka University Medical Center and Faculty of Health Sciences, Ben-Gurion University of the Negev, Be'er Sheva, Israel; ^2^Research Department, Tikun Olam - Cannbit Pharmaceuticals, Tel Aviv, Israel; ^3^Institute for Drug Research, Medical Faculty Hebrew University, Jerusalem, Israel; ^4^Geriatric Department, Hadarim Nursing Home, Naan, Israel; ^5^Department of Gastroenterology and Hepatology, Meir Medical Center, Kfar Saba and Faculty of Medicine, Tel Aviv University, Tel Aviv, Israel; ^6^Department of Anesthesia, Critical Care and Pain Medicine, Harvard Medical School, Beth Israel Deaconess Medical Center, Boston, MA, United States

**Keywords:** cannabis, cannabidiol (CBD), tetrahydrocannabinol (THC), prospective, cohort, pain, quality of life, adherence

## Abstract

**Background:**

Despite the absence of rigorous prospective studies, there has been an increase in the use of cannabis-based medicinal products. During the study period, the use of medical cannabis in Israel was tightly regulated by national policy. Through a prospective study of approximately 10,000 patients, we aimed to characterize the medical cannabis patient population as well as to identify treatment adherence, safety, and effectiveness.

**Methods and Findings:**

In this study of prescribed medical cannabis patients, adherence, safety, and effectiveness were assessed at 6 months. Treatment adherence was assessed by the proportion of patients purchasing the medication out of the total number of patients (excluding deceased cases and patients transferred to another cannabis clinic). Safety was assessed by the frequency of the side-effects, while effectiveness was defined as at least moderate improvement in the patient condition without treatment cessation or serious side-effects. The most frequent primary indications requiring therapy were cancer (49.1%), followed by non-specific pain (29.3%). The average age was 54.6 ± 20.9 years, 51.1% males; 30.2% of the patients reported prior experience with cannabis. During the study follow-up, 1,938 patients died (19.4%) and 1,735 stopped treatment (17.3%). Common side-effects, reported by 1,675 patients (34.2%), were: dizziness (8.2%), dry mouth (6.7%), increased appetite (4.7%), sleepiness (4.4%), and psychoactive effect (4.3%). Overall, 70.6% patients had treatment success at 6 months. Multivariable logistic regression analysis revealed that the following factors were associated with treatment success: cigarette smoking, prior experience with cannabis, active driving, working, and a young age. The main limitation of this study was the lack of data on safety and effectiveness of the treatment for patients who refused to undergo medical assessment even at baseline or died within the first 6 months.

**Conclusions:**

We observed that supervised medical-cannabis treatment is associated with high adherence, improvement in quality of life, and a decrease in pain level with a low incidence of serious adverse events.

## Introduction

In recent years, there has been an increase in the use of cannabis-based products for a wide range of medical purposes, despite a lack of sufficient scientific evidence supporting cannabis therapies (1). Non-purified products of the cannabis plant are the most frequently consumed by cannabis users (2), and contain three families of components, terpenes, flavonoids, and cannabinoids (3). Delta-9-tetrahydrocannabinol (THC) and cannabidiol (CBD) are the two most common cannabinoids found in the cannabis plant (4). THC is the primary psychoactive ingredient (5) and has shown therapeutic benefit for pain, nausea, and sleep (1). CBD is non-intoxicating at medically relevant doses (4); and when combined with THC, may counterbalance the psychoactivity of THC (6), while increasing THC tolerance (7). Cannabidiol has anti-inflammatory, neuroprotective, antipsychotic, anxiolytic, and antidepressant properties (8).

Although the UK has begun to develop a registry of medical cannabis patients (9), rigorous observational studies and prospective clinical trials have yet to be undertaken and most of the available data is derived from surveys of cooperating users. These surveys are usually limited in scope, retrospective, and rarely collect data on variables beyond basic demographic elements, comorbidities, modes of consumption, and overall satisfaction (2, 6–8, 10–23).

Medical cannabis is now available in many countries, where it is primarily used for its analgesic effect (2, 10–12, 15, 16, 18, 24–26). In 2007, the Israeli Ministry of Health (IMOH) began issuing licenses for the use of cannabis for patients with specific indications, including: nausea and vomiting due to chemotherapy treatment, cancer associated pain, inflammatory bowel disease (IBD), neuropathic pain, fibromyalgia, cachexia in AIDS (acquired immunodeficiency syndrome) patients, multiple sclerosis (MS), Parkinson's disease (PD), Tourette syndrome, epilepsy, autism, and post-traumatic stress disorder (PTSD). A physician can recommend medical cannabis under one of the indications approved by the IMOH, only following the exhaustion of all traditional medications options. A license to receive medical cannabis may then be granted to a patient, and that license associated with a specialized clinic. Because of this aspect of the regulation, we could assess the effect of treatment of all patients enrolled in the clinic, where all are tested, with no collection bias. As more countries legalize medical cannabis use and some legalize recreational use, accrual of scientific data on treatment adherence, safety, and effectiveness is essential. The first step in this process should be based on the evaluation of rigorously accumulated observational data. Therefore, the aim of this study is to prospectively assess the characteristics of the patient population and evaluate adherence, safety, and effectiveness of medical cannabis in a tightly regulated environment.

## Materials and Methods

### Study Population and Treatment Program

This study was conducted based on clinical data collected as part of the treatment program in Israel's largest cannabis clinic. The study included all patients who received a medical cannabis treatment license through the clinic between March 2015 and February 2018. According to the clinic's standard protocols, each patient had the option to receive a 45-min intake session. This session was designed so that the attending nurse could assess the patient's complete medical history, advise on a suitable selection from cannabis chemovars of varying cannabinoid concentrations, and to explain the recommended method of administration and titration process. Six months after the initiation of treatment, willing patients participated in a telephone interview to assess changes in symptom intensity and side-effects. If needed, the nurse recommended treatment adjustments.

We have published data based on this database in four previous studies, on cancer patients (27), patients over the age of 65 (28), fibromyalgia patients (29), and children with autism (30). There is a certain overlap between the patients presented in these studies and the current study, especially in the cancer patient study, published in 2018, which included 1,248 patients, and the study on patients over the age of 65 which included 901 patients, where we assessed the effect of at least 6 months' active medical cannabis treatment. However, in this study, we expanded the focus to all indications for cannabis treatment, over a prolonged recruitment period. Moreover, in this study, we analyzed only patients who answered the intake questionnaire after receiving a new cannabis treatment license, so that the baseline represents a pre-medical cannabis treatment state.

### Outcome Measures

#### Patient Characterization

The characteristics of the medical cannabis patient population were analyzed as one large group and divided based on the main indication for medical cannabis treatment of each patient. We included all patients who filled the intake questionnaire, i.e., 85.7% of patients who initiated treatment.

#### Adherence

Patient adherence to the treatment regimen was assessed based on actual refill orders, calculated as the proportion of patients purchasing the medication out of the total number of patients at both 1 month and at 6 months treatment duration, excluding deceased cases and patients transferred to another cannabis clinic. Treatment adherence was assessed in all patients, and not only in patients answering the questionnaire.

#### Safety

Side-effects were assessed at 6 months by first asking the patient “Have you experienced side-effects due to the use of cannabis?” If the answer was “yes,” the patient was asked to specify the side-effects via a free text response coded as a predefined list of the common side-effects. Patients were asked details of incidence (rarely, sometimes, often, or always), duration (several minutes, half hour, several hours, all day), and severity (1–10) of any reported side-effects. We included all active and inactive patients that answered this 6-month follow-up questionnaire.

#### Effectiveness

For analysis of treatment effectiveness, we used the global assessment approach where patients were asked at 6 months: “How would you rate the general effect of cannabis on your condition?” The seven response options were: significant, moderate, or slight improvement, no change, slight, moderate, or significant deterioration. For the primary effectiveness endpoint analysis, we selected a conservative approach, and so treatment success was defined as (a) at least moderate or significant improvement in the patient's condition and (b) none of the following: treatment cessation or serious side-effects defined as 9–10 on severity scale and incidence of often or always. We included all patients who discontinued treatment during the first 6 months of treatment and all patients who remained in active treatment during this period and answered the 6-month follow-up questionnaire. All patients who discontinued treatment and patients who were lost to follow-up were classified as a treatment failure.

For effectiveness in specific parameters like pain, quality of life (QOL), and change in concomitant medication consumption, we analyzed patients who answered the relevant question in both time points (before treatment and after 6 months of active treatment). We used a numeric rating scale to assess pain level on an 11-point scale (0 = no pain, 10 = worst pain imaginable) (31), and a Likert scale to assess QOL (very poor, poor, neither poor nor good, good, very good) (32). We analyzed the changes over time in the pain and QOL rating scales of each patient as a paired comparison.

Furthermore, patients were asked, both at intake and in the 6-months follow-up questionnaire, to report all the prescribed medications they regularly take, dose, and number of administrations per day. The medications were sorted in classes according to the international ATC (Anatomical Therapeutic Chemical) drugs classes distribution to assess changes over time.

A significant principle in cannabis treatment is to map all the symptoms the patient suffers from, and to match expectations with the patient on the symptoms that usually are improved with cannabis products (pain, sleep disturbances, nausea and vomiting, spasticity, depression, and others); these are the treatment goals. The first step is to focus on the symptom that is most bothersome—and to match a product for that symptom. The therapeutic dose is a dose that achieves a balance between maximum reduction of target symptom and a minimum of side-effects. To reach the therapeutic dose, the patient must undergo a process of titration. After an improvement in the main symptom, we may incorporate another product and another treatment goal. The recommendation for chemovars and products was based on the experience accumulated at the clinic regarding which product has the highest effectiveness rate for a specific symptom. The products are based on chemovars (sativa or indica dominant, high THC, high CBD, or balanced) and consumption method (flowers for inhalation or smoking, oil under the tongue, and capsules).

### Statistical Analysis

Continuous variables with normal distribution were presented as means with standard deviation. Ordinary variables or continuous variables with non-normal distribution were presented as medians with an interquartile range (IQR). Categorical variables were presented as counts and percent of the total.

We used a *t*-test for the analysis of continuous variables with normal distribution, Mann–Whitney U-test whenever parametric assumptions could not be satisfied, and χ^2^-test for categorical variables. For paired comparisons, we have used paired *t*-test, non-parametric Wilcoxon signed rank test, and χ^2^-test for dependent variables. We used multivariable logistic regression analysis for the factors associated with the treatment success to control possible confounders. We have included the following baseline variables into the models based on clinical considerations: age, gender, weight, indication for cannabis treatment, presence of pain, number of chronic medications, hospitalization in the past 6 months, employment, car use, previous experience with cannabis, cigarette smoking, QOL, and concerns about cannabis treatment as reflected in the intake form. The final model was selected according to the model characteristic, evaluated by calculating the c-statistic, in addition to choosing the minimal −2 log likelihood of each model.

Results are displayed as odds ratios with 95% confidence interval. *P*-value < 0.05 was considered statistically significant. All analyses were performed at the Clinical Research Center, Soroka University Medical Center, Beer-Sheva, Israel, using IBM SPSS version 22 (SPSS, Chicago, IL). The Sponsor was not involved in the data analysis.

### Ethics Approval and Consent to Participate

This study was approved by the IRB at the Soroka University Medical Center, Beer-Sheva, Israel, study number: SCRC-0415-15. Although data was collected prospectively, the need for informed consent was waived due to the non-intervention nature of the study and the retrospective data analysis.

## Results

### Patient Population

**Figure 1:**
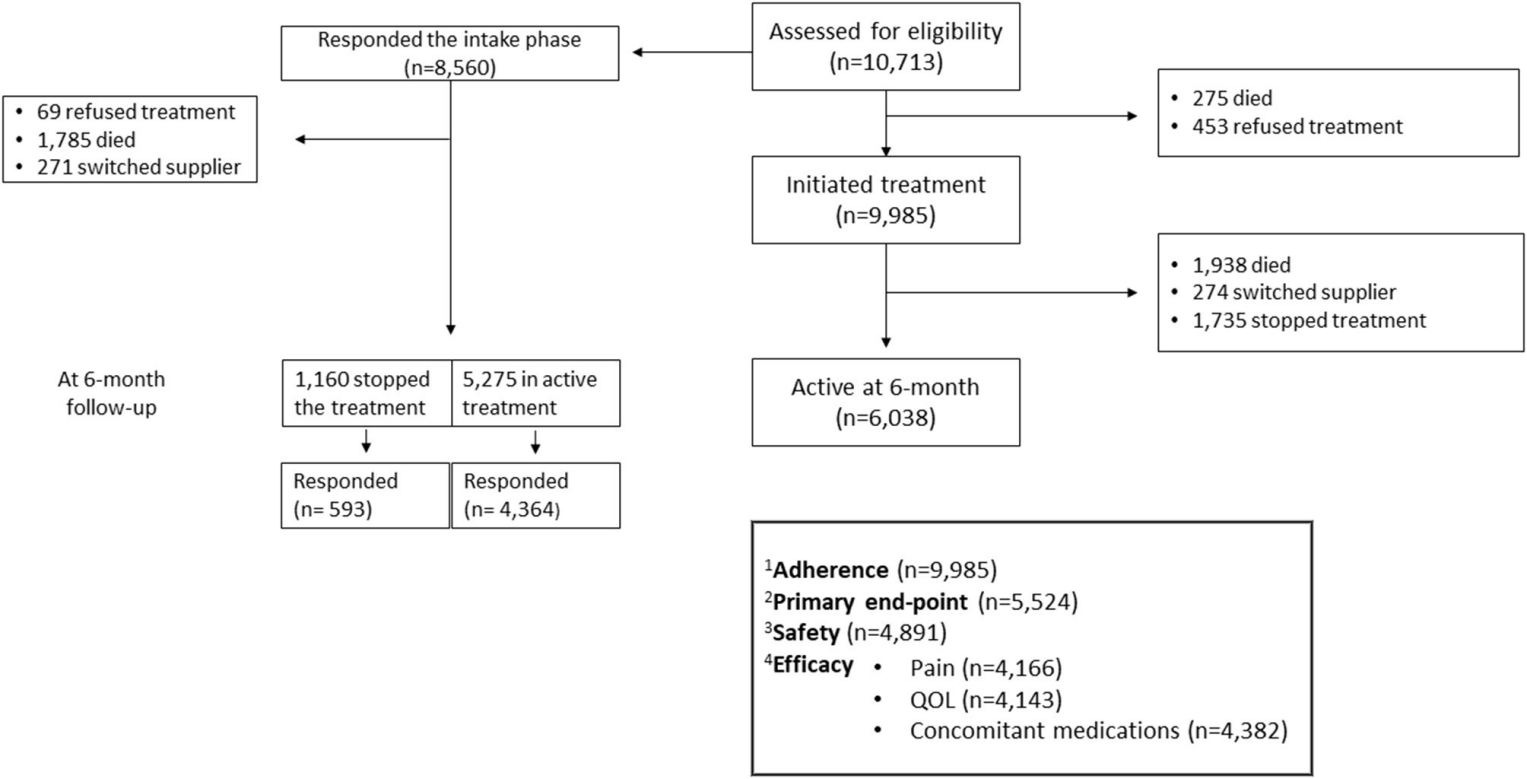
The study population. Detailed description of the patients included into the adherence assessment, primary endpoint assessment, and the safety and effectiveness analysis population. ^1^Adherence analysis was performed on all patients who initiated treatment. ^2^Primary end-point analysis was performed on patients who responded the intake questionnaire and: responded to the 6-month follow-up questionnaire and all patients who stopped the treatment. ^3^Safety analysis was performed on all patients who responded to the side-effect section of the 6-month follow-up questionnaire, both active patients and responders that stopped the treatment. ^4^Efficacy analysis was performed on patients who answered the specific chapter at the intake session and at the 6-month follow-up questionnaire (active patients only).

During 3 years of study period, 10,713 subjects received their first cannabis treatment license: 2.6% died before starting treatment, 4.2% opted not to receive the treatment, and 9,985 patients (93.2%) initiated treatment. Out of these, 8,560 (85.7%) responded to the intake questionnaire (see Figure 1 for a detailed flow diagram and for the cohort in each of the outcome analyses). The patients, mean age 54.6 years, 51.1% men, received a cannabis treatment license for the following indications: cancer (49.1%: chemotherapy related symptoms 23.5% and pain related treatment 25.5%), non-specific pain (29.4%), PTSD (6.4%), autism (3.6%), epilepsy (2.7%), PD (2.5%), IBD (2.2%), MS (0.9%), compassionate care (0.6%), Tourette syndrome (0.6%), and others (1.9%) (full demographic characteristics are presented in Table 1). Each patient has one indication for the cannabis treatment license, but usually more than one medical condition. Supplementary Table 1 shows the full list of comorbidities with the disease duration: 52.1% had cancer, 18.7% suffered from pain, 14.0% suffered from hypertension, and 10.6% had diabetes. The median disease duration was 4 years (range 1–21).

**Table 1:** Patient's demographic characteristics.

	**Total** **(8,560)**	**Cancer** **(4,205)**	**Non-specific pain** **(2,515)**	**PTSD** **(551)**	**Autism** **(311)**	**Epilepsy** **(232)**	**PD** **(215)**	**IBD** **(190)**	**MS** **(79)**	**Compassionate** **(55)**	**Tourette syndrome** **(48)**	**Others** **(159)**
Mean age (SD)	54.6 (20.9)	61.1 (16.2)	57.0 (18.7)	41.4 (13.7)	12.2 (6.1)	16.6 (13.4)	71.9 (9.6)	38.0 (14.4)	47.4 (11.3)	35.4 (27.8)	31.4 (13.2)	44.0 (26.7)
Gender (male), no. (%)	4,379 (51.1)	1,908 (45.4)	1,287 (51.2)	382 (69.3)	261 (83.9)	122 (52.6)	124 (57.7)	103 (54.2)	34 (43.0)	35 (63.6)	36 (75.0)	87 (54.7)
Working (Yes), no. (%)	2,017 (23.5)	693 (16.4)	765 (30.4)	266 (48.2)	3 (0.9)	36 (15.5)	17 (7.9)	122 (64.2)	35 (44.3)	20 (36.3)	27 (56.2)	32 (20.1)
Driving a car (Yes), no. (%)	4,165 (48.6)	2,008 (47.7)	1,403 (55.7)	389 (70.5)	0 (0.0)	5 (2.1)	55 (25.5)	161 (84.7)	53 (67.0)	13 (23.6)	29 (60.4)	49 (30.8)
Median number of hospitalization days in the past 6 months (IQR)	0 (0–7)	3 (0–14)	0 (0–0)	0 (0–0)	0 (0–0)	0 (0–1.7)	0 (0–0)	0 (0–2)	0 (0–0)	0 (0–3.5)	0 (0–0)	0 (0–2.5)
Median number of medications (IQR)	3 (1–6)	3 (1–6)	4 (2–7)	2 (0–4)	1 (0–2)	3 (2–4)	7 (4–9)	2 (1–3)	3 (1.5–5)	2 (1–4)	1 (0–3)	3 (1–5.5)
Previous experience with cannabis (Yes), no. (%)	2,590 (30.2)	927 (22.3)	1,010 (40.7)	356 (65.7)	17 (5.5)	22 (9.6)	54 (25.2)	95 (50.5)	35 (45.5)	4 (7.5)	26 (54.2)	44 (27.8)
Cigarette smoking (Yes), no. (%)	2,081 (24.3)	743 (17.6)	904 (35.9)	272 (49.3)	1 (0.3)	16 (6.8)	22 (10.2)	50 (26.3)	26 (32.9)	4 (7.2)	16 (33.3)	27 (16.9)
Median pain scale 0–10 (IQR)	8 (4–10)	7 (3–9)	9 (8–10)	5 (0–8)	0 (0–0)	0 (0–0)	8 (5.7–9)	8 (7–9)	8 (6–10)	0 (0–0)	0 (0–8)	5 (0–9)

*Characteristics are for all patients and per medical indication for the cannabis license of each patient. PTSD, post-traumatic stress disorder; PD, Parkinson's disease; IBD, inflammatory bowel disease; MS, multiple sclerosis*.

Out of the patients responding to the intake questionnaire, 7,056 reported on regular consumption of prescription medications (82.4%). The main families of drugs used were: opioids (32.5%), anti-depressants (29.9%), anti-epileptics (26.2%), and drugs for peptic ulcer and gastroesophageal reflux disease (23.2%) (Supplementary Table 2).

At baseline, patients reported an average of 9.8 ± 7.4 symptoms. Table 2 shows the prevalence of symptoms at the time treatment was initiated: 79.1% reported sleep disturbances, 77.1% pain, and 55.6% reported weakness and fatigue.

**Table 2:** Symptoms prevalence at intake.

**Symptom** **no.** **(%)**	**Total** **(8,560)**	**Cancer** **(4,205)**	**Non-specific pain** **(2,515)**	**PTSD** **(551)**	**Autism** **(311)**	**Epilepsy** **(232)**	**PD** **(215)**	**IBD** **(190)**	**MS** **(79)**	**Compassionate** **(55)**	**Tourette syndrome (48)**	**Others** **(159)**
Sleep disturbances	6,772 (79.1)	3,279 (78.0)	2,152 (85.6)	518 (94.0)	180 (57.9)	110 (47.4)	163 (75.8)	147 (77.4)	57 (72.2)	42 (76.4)	27 (56.3)	107 (67.3)
Pain	6,567 (77.1)	3,173 (76.0)	2,445 (99.4)	308 (55.8)	3 (1.0)	39 (17.3)	179 (85.6)	178 (95.2)	68 (88.3)	12 (22.6)	20 (43.5)	142 (62.6)
Weakness and fatigue	4,756 (55.6)	2,903 (69.0)	1,158 (46.0)	302 (54.8)	8 (2.6)	66 (28.4)	93 (43.3)	102 (53.7)	42 (53.2)	28 (50.9)	14 (29.2)	58 (36.5)
Digestion problems	4,071 (47.6)	2,458 (58.5)	962 (38.3)	199 (36.1)	17 (5.5)	54 (23.3)	100 (46.5)	179 (94.2)	21 (26.6)	21 (38.2)	7 (14.6)	61 (38.4)
Anxiety	3,492 (41.7)	1,737 (42.0)	883 (36.4)	472 (87.1)	110 (35.9)	40 (17.5)	86 (40.0)	55 (28.9)	20 (26.0)	24 (43.6)	16 (37.2)	49 (31.4)
Restlessness	3,090 (36.9)	1,253 (30.3)	863 (35.5)	349 (64.4)	267 (87.3)	90 (39.5)	77 (35.8)	65 (34.2)	19 (24.7)	37 (67.3)	17 (39.5)	53 (34.0)
Depression	3,695 (44.1)	1,839 (44.5)	1,122 (46.2)	439 (81.0)	7 (2.3)	30 (13.2)	102 (47.4)	47 (24.7)	23 (29.9)	21 (38.2)	11 (25.6)	54 (34.6)
Lack of appetite	3,694 (44.1)	2,350 (56.8)	803 (33.1)	224 (41.3)	14 (4.6)	46 (20.2)	68 (31.6)	106 (55.8)	18 (23.4)	18 (32.7)	5 (11.6)	42 (26.9)
Nausea	3,023 (36.1)	2,162 (52.3)	530 (21.8)	154 (28.4)	3 (1.0)	16 (7.0)	25 (11.6)	96 (50.5)	7 (9.1)	11 (20.0)	2 (4.7)	17 (10.9)
Movement limitation	2,961 (35.4)	1,303 (31.5)	1,159 (47.7)	96 (17.7)	18 (5.9)	97 (42.5)	120 (55.8)	26 (13.7)	43 (55.8)	17 (30.9)	5 (11.6)	77 (49.4)
Paresthesia	2,721 (32.5)	1,337 (32.3)	1,085 (44.7)	129 (23.8)	1 (0.3)	8 (3.5)	45 (20.9)	28 (14.7)	49 (63.6)	8 (14.5)	7 (16.3)	24 (15.4)
Spasticity	2,460 (29.4)	992 (24.0)	924 (38.0)	138 (25.5)	3 (1.0)	51 (22.4)	163 (75.8)	41 (21.6)	60 (77.9)	14 (25.5)	8 (18.6)	66 (42.3)
Dizziness	2,005 (23.9)	1,201 (29.0)	531 (21.9)	140 (25.8)	1 (0.3)	15 (6.6)	35 (16.3)	38 (20.0)	20 (26.0)	5 (9.1)	4 (9.3)	15 (9.6)
Agitation	1,981 (23.7)	814 (19.7)	554 (22.8)	248 (45.8)	190 (62.1)	39 (17.1)	24 (11.2)	34 (17.9)	15 (19.5)	26 (47.3)	11 (25.6)	26 (16.7)
Burning sensation	1,659 (19.8)	760 (18.4)	704 (29.0)	99 (18.3)	2 (0.7)	5 (2.2)	16 (7.4)	30 (15.8)	19 (24.7)	6 (10.9)	2 (4.7)	16 (10.3)
Dry mouth	1,635 (19.5)	1,072 (25.9)	363 (14.9)	108 (19.9)	1 (0.3)	9 (3.9)	34 (16.2)	13 (6.9)	8 (10.4)	9 (16.4)	2 (4.7)	16 (10.3)
Headache	1,574 (18.8)	788 (19.1)	517 (21.3)	151 (27.9)	2 (0.7)	22 (9.6)	15 (7.0)	35 (18.4)	12 (15.6)	5 (9.1)	8 (18.6)	19 (12.2)
Respiratory problems	1,537 (18.4)	955 (23.1)	376 (15.5)	79 (14.6)	4 (1.3)	31 (13.6)	21 (9.8)	22 (11.6)	7 (9.1)	11 (20.0)	2 (4.7)	29 (18.6)
Cognitive impairment	1,266 (15.1)	574 (13.9)	282 (11.6)	97 (17.9)	91 (29.7)	113 (49.6)	26 (12.1)	8 (4.2)	10 (13.0)	28 (50.9)	3 (7.0)	34 (21.8)
Tremor	1,203 (14.4)	535 (12.9)	323 (13.3)	95 (17.5)	1 (0.3)	35 (15.4)	154 (71.6)	8 (4.2)	13 (16.9)	11 (20.0)	1 (2.3)	27 (17.3)
Pruritus	1,198 (14.3)	639 (15.5)	369 (15.2)	102 (18.8)	3 (1.0)	9 (3.9)	13 (6.0)	29 (15.3)	4 (5.2)	12 (21.8)	7 (16.3)	11 (7.1)
Rage attacks	1,191 (14.2)	371 (9.0)	262 (10.8)	220 (40.6)	224 (73.2)	37 (16.2)	9 (4.2)	12 (6.3)	8 (10.4)	24 (43.6)	4 (9.3)	20 (12.8)
Visual impairment	937 (10.9)	530 (12.8)	242 (10.0)	49 (9.0)	4 (1.3)	39 (17.1)	18 (8.4)	6 (3.2)	9 (11.7)	14 (25.5)	3 (7.0)	22 (14.1)

*PTSD, post-traumatic stress disorder; PD, Parkinson's disease; IBD, inflammatory bowel disease; MS, multiple sclerosis*.

At baseline, a total of 15.0% reported having concerns over the initiation of cannabis treatment. The most common concerns were potential side-effects (3.5%), lack of knowledge regarding the effect (1.2%), lack of effect (0.8%), addiction (0.8%), loss of control (0.7%), worsening medical condition (0.5%), cannabis being an illicit drug (0.5%), and the “high” effect (0.4%). For comparison between patients with and without cannabis previous experience, please refer to Supplementary Table 3.

### Adherence

Adherence was assessed for all patients who initiated treatment in the cannabis clinic. After1 month, of the 9,985 patients who started the treatment, 4.8% died, 5.2% stopped treatment, and 0.3% switched to a different cannabis supplier, while 89.7% continued active treatment. Of those who continued active treatment, 6,699 (74.8%) responded to the questionnaire. Of them, 2,562 patients (38.2%) experienced side-effects or reported that the cannabis did not improve their condition during the first month of the treatment and needed the advice and guidance of a nurse to adjust the dose or the treatment. At 6 months of 7,773 patients, 6,038 (77.7%) remained in active treatment (excluding 19.4% patients who died and 2.7% who switched to a different cannabis supplier).

### Safety Analysis

Of the 4,891 patients who responded to the side-effect follow-up questionnaire, 1,675 patients (34.2%) reported experiencing at least one side-effect. The most common were dizziness (8.2%), dry mouth (6.7%), increased appetite (4.7%), sleepiness (4.4%), and psychoactive effects (4.3%) (Table 3). This analysis included all active patients and patients who discontinued the cannabis treatment.

**Table 3:** Frequency of adverse events at the 6-months follow-up questionnaire.

**Side-effects experienced due to the use of cannabis (4,891), no. (%)**	**Total responses, no. (%)**
**Have you experienced side-effects due to the use of cannabis? (Yes)**	1,675 (34.2)
**Physiological**	
Dizziness	399 (8.2)
Dry mouth	329 (6.7)
Increased appetite	232 (4.7)
Sleepiness	217 (4.4)
Nausea	143 (2.9)
Weakness	141 (2.9)
Drop in sugar	105 (2.1)
Headaches	83 (1.7)
Cough	75 (1.5)
Vomiting	55 (1.1)
Burning sensation in throat	48 (1.0)
Red/irritated eyes	43 (0.9)
Increased heart rate	41 (0.8)
Stomachache	28 (0.6)
Drop in blood pressure	27 (0.6)
Decreased appetite	20 (0.4)
Blurred vision	19 (0.4)
Tremor	14 (0.3)
Sleep disturbance	12 (0.2)
Difficulty breathing	12 (0.2)
Itching	10 (0.2)
Slurred speech	10 (0.2)
Diarrhea	10 (0.2)
Constipation	6 (0.1)
Chills	2 (0.04)
**Cognitive**	
Psycho-active effects (feeling “high”)	208 (4.3)
Confusion and disorientation	83 (1.7)
Restlessness	69 (1.4)
Hallucinations	61 (1.2)
Decreased concentration	50 (1.0)
Decreased memory	41 (0.8)
Fear	38 (0.8)
Anxiety	17 (0.3)
Gloominess	13 (0.3)
Nervousness	10 (0.2)
Apathy	5 (0.1)
Other	66 (1.3)

Increased appetite was reported as a side effect by 232 patients (4.7% overall and 2.0% as a lone side effect); 36.6% of them received their cannabis license for pain indication, 34.4% for cancer, 11.7% for PTSD, 4.3% for Crohn's and colitis, and 3.9% for autism.

Of those responding to the side-effects chapter, 2.9% reported nausea. This rate varied between different chemovars in the interval of 1.2–3.8%, with THC rich indica chemovar “Dorit” being the highest.

### Primary Effectiveness Outcome

The primary effectiveness outcome was assessed for all respondents to the intake questionnaires except for patients refusing treatment (69), deceased patients (1,785), patients switching to other providers (271), and active patients who did not respond to the follow-up questionnaire (911). Thus, the primary effectiveness outcome was assessed for 5,524 of the 8,560 patients responding to the intake questionnaire (64.5%, Figure 1). Overall, 3,902 (70.6%) patients out of 5,524 experienced treatment success. Multivariable analysis revealed the following factors as associated with treatment success: cigarette smoking (O.R 2.4, 95% C.I 2.0–2.2), prior experience with cannabis (O.R 2.1, 95% C.I 1.8–2.5), driving (O.R 1.3, 95% C.I 1.1–1.5), employment (O.R 1.3, 95% C.I 1.0–1.5), and young age (O.R 0.9, 95% C.I 0.9–0.9), (Table 4).

**Table 4:** Logistic regression multivariable analysis of factors associated with treatment success after 6 months.

	**Odds ratio**	**95% Confidence interval**	***P*-value**
Current cigarette smoking vs. non-smokers	2.40	2.01–2.86	<0.001
Previous experience with cannabis vs. no previous experience	2.16	1.83–2.54	<0.001
Active drivers vs. non-drivers	1.36	1.17–1.56	<0.001
Employed vs. unemployed	1.30	1.08–1.55	<0.01
Mean age	0.98	0.98–0.99	<0.001

*Treatment success was defined as at least a moderate or significant improvement in the patient's condition and none of the following: cessation of treatment or serious side-effects*.

Of 4,364 patients who answered to the 6-months follow-up questionnaire, the most common chemovar used was an 18% THC indica (Erez, 2,551 patients, 55.7%). This chemovar was most often consumed by smoking or vaporization (1,306 patients), at an average dried flowers weight of 0.3 g (54 mg THC) per administration, and a frequency of 3.4 administrations per day. A total of 935 patients consumed Erez sublingual oil 300 mg THC/10 ml, consuming an average dose of 5.7 mg THC per administration, and a frequency of 2.4 administrations per day (further description in Table 5).

**Table 5:** Cannabis consumption characteristics.

**Chemovar**	**Description**	**Number of patients (%)**	**Most common consumption time**	**Most common methods of administration**	**Average number of administration per day**	**Average dose per administration**	**Average cannabinoid dose per administration**
Erez	18% THC, 0% CBD Indica	2,551 (55.7%)	Evening (1,590 patients) and night (1,030 patients)	1,306 smoking or evaporation	3.4	0.3 g	54 mg THC
				935 sublingual oil (300 mg THC/10 ml)	2.4	3.8 Drops^*^	5.7 mg THC
Alaska	18% THC, 0% CBD Sativa	2,144 (46.8%)	Morning (1,382 patients) and afternoon (1,388 patients)	1,870 smoking or evaporation	4.3	0.3 g	54 mg THC
				221 sublingual oil (300 mg THC/10 ml)	3.3	3.5 Drops^*^	5.2 mg THC
Avidekel	15% CBD, 0.5% THC Indica	1,451 (31.7%)	Morning (908 patients) and afternoon (753 patients)	210 smoking or evaporation	4.2	0.26 g	39 mg CBD
				976 sublingual oil (300 mg CBD/10 ml)	2.5	4.5 Drops^*^	6.7 mg CBD

* *One drop is equivalent to ~0.04 ml*.

The most improved symptoms were rage attacks (decrease of 91.5%), restlessness (89.5%), sleep disturbances (89.1%), and nausea (88.9%). For more information about the changes in the specific symptoms after 6 months, please refer to Supplementary Table 4. Of the patients reporting nausea at intake and responding to the follow-up questionnaire, 30.5% reported that they no longer suffer from nausea, 58.4% reported that the symptom improved, 10.0% reported no change in nausea they experience, and 1.1% of patients reported deterioration in the nausea they experienced.

**Figure 2:**
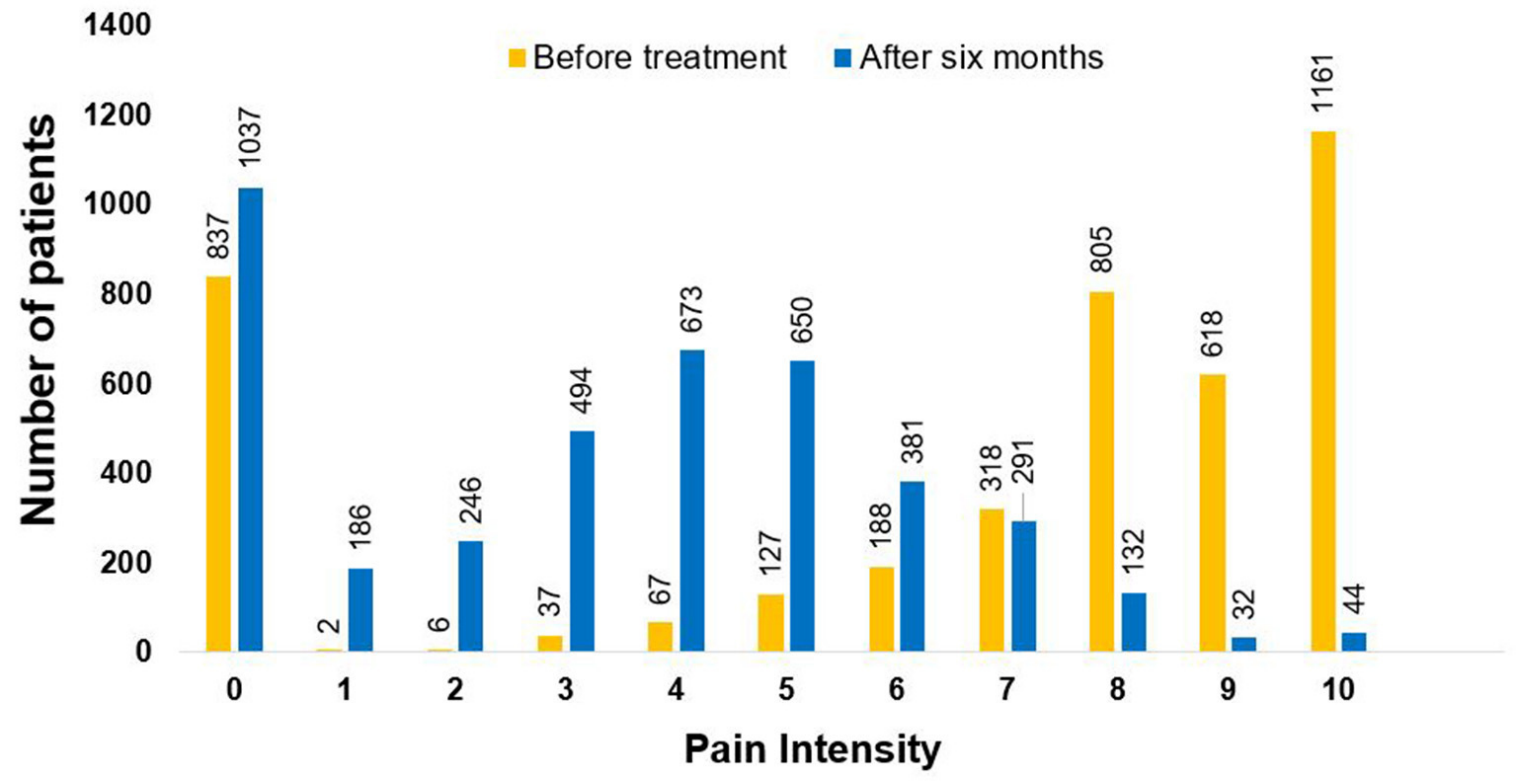
Assessment of pain intensity. Pain intensity was assessed on a 0–10 scale, before and after 6 months of cannabis therapy (*p* < 0.001). The assessment was made on 4,166 patients who responded to this question at the two time points. Pain level was measured on an 11-point scale (0 = no pain, 10 = worst pain imaginable).

Pain intensity was assessed both at intake and at 6 months in 4,166 patients. Prior to treatment initiation, 62.0% of patients reported their pain at between 8 and 10, while only 5.0% reported this intensity at 6 months (*p* < 0.001, Figure 2); 7.3% of the patients demonstrated deterioration in their pain scale. In 17.8%, the level of pain did not change while in 74.7% it improved, of which 64.3% of patients showed an improvement of 30% or more in their reported pain intensity and 47.2% reported an improvement of 50% or more in their pain intensity. In 1,580 patients, only under the pain indication, 85.9% experienced an improvement of 30% or more, and 59.3% an improvement of 50% or more in their VAS pain scale.

Pain intensity was assessed both at intake and at 6 months in 4,166 patients. Prior to treatment initiation, 62.0% of patients reported their pain at between 8 and 10, while only 5.0% reported this intensity at 6 months (*p* < 0.001, Figure 2); 7.3% of the patients demonstrated deterioration in their pain scale. In 17.8%, the level of pain did not change while in 74.7% it improved, of which 64.3% of patients showed an improvement of 30% or more in their reported pain intensity and 47.2% reported an improvement of 50% or more in their pain intensity. In 1,580 patients, only under the pain indication, 85.9% experienced an improvement of 30% or more, and 59.3% an improvement of 50% or more in their VAS pain scale.

**Figure 3:**
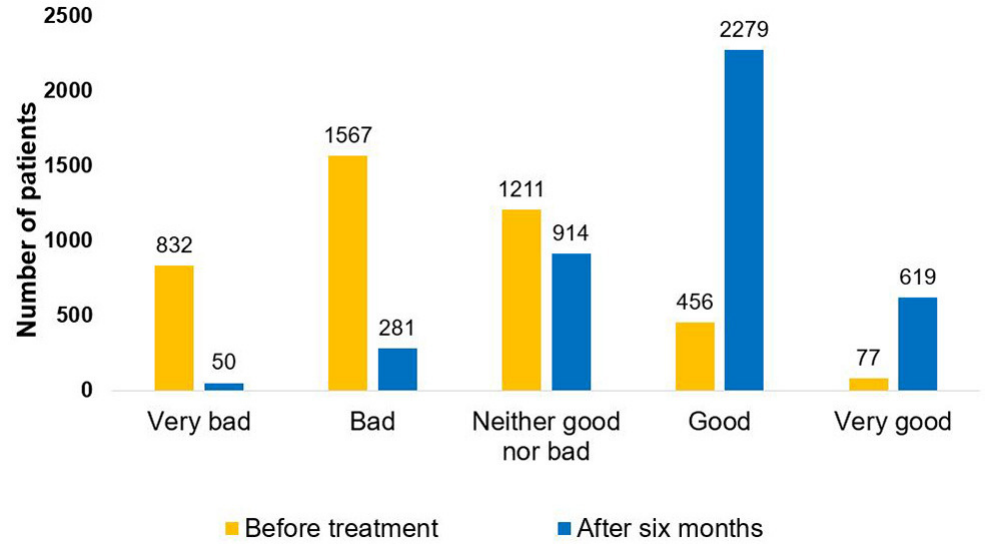
Quality of life assessment. Quality of life was assessed prior to and 6 months after initiation of cannabis treatment (*p* < 0.001). Assessment was done on 4,143 patients who responded twice to this question.

Quality of life (QOL) was assessed both at intake and at 6 months in 4,143 patients. While only 12.9% of patients reported good QOL prior to treatment initiation, 69.9% reported good QOL at 6 months (*p* < 0.001, Figure 3).

Concomitant medications consumption was evaluated both at the intake and in the follow-up questionnaires in 3,544 patients. The most reduced medications classes were opioids (52.5%), other analgesics and antipyretics (39.2%), anti-psychotics (36.9%) anti-epileptics (35.7%), and hypnotics and sedatives (35.3%) (Table 6).

**Table 6:** Prescription medication use at 6 months follow-up.

**Drug class**	**Total** **responders**	**Same dose**	**Stopped consuming this medication**	**Dose decreased**	**Dose increased**	Other^*^	**Patients who started taking a drug that was not taken during intake session**
Opioids	1,216	553 (45.5)	472 (38.8)	167 (13.7)	24 (2.0)	3 (0.2)	63
Antidepressants	1,232	815 (66.2)	310 (25.2)	83 (6.7)	24 (1.9)	3 (0.2)	93
Antiepileptics	1,098	680 (61.9)	282 (25.7)	110 (10.0)	26 (2.4)	0 (0.0)	61
Drugs for peptic ulcer and gastroesophageal reflux disease (GERD)	713	568 (79.7)	119 (16.7)	21 (2.9)	5 (0.7)	2 (0.3)	61
Antithrombotic agents	697	606 (86.9)	79 (11.3)	11 (1.6)	1 (0.1)	2 (0.3)	38
Anxiolytics	657	496 (75.5)	109 (16.6)	46 (7.0)	6 (0.9)	0 (0.0)	17
Lipid modifying agents	679	565 (83.2)	102 (15.0)	9 (1.3)	3 (0.4)	2 (0.3)	22
Hypnotics and sedatives	600	386 (64.3)	166 (27.7)	46 (7.7)	2 (0.3)	3 (0.5)	27
Other analgesics and antipyretics	471	285 (60.5)	141 (29.9)	44 (9.3)	1 (0.2)	2 (0.4)	22
Ace-inhibitors	350	298 (85.1)	39 (11.1)	11 (3.1)	2 (0.6)	5 (1.4)	10
Blood glucose lowering agents, excluding insulin	324	270 (83.3)	38 (11.7)	15 (4.6)	1 (0.3)	0 (0.0)	21
Selective calcium channel blockers with mainly vascular effects	299	258 (86.3)	37 (12.4)	3 (1.0)	1 (0.3)	2 (0.7)	6
Corticosteroids for systemic use	242	159 (65.7)	65 (26.9)	17 (7.0)	1 (0.4)	1 (0.4)	21
Beta blocking agents	255	220 (86.3)	27 (10.6)	7 (2.7)	1 (0.4)	1 (0.4)	10
Antipsychotics	276	169 (61.2)	64 (23.2)	38 (13.8)	5 (1.8)	0 (0.0)	21
Thyroid preparations	248	222 (89.5)	16 (6.5)	8 (3.2)	2 (0.8)	1 (0.4)	12

*Change in consumption of main medications families that were consumed regularly during intake session in active patients responded to the 6-months follow-up questionnaire, for all the patients and per indication. PTSD, post-traumatic stress disorder; IBD, inflammatory bowel disease; PD, Parkinson's disease; MS, multiple sclerosis*.

* *Other: the two most reported answers under this rubric were: I do not remember and as needed*.

**Figure 4:**
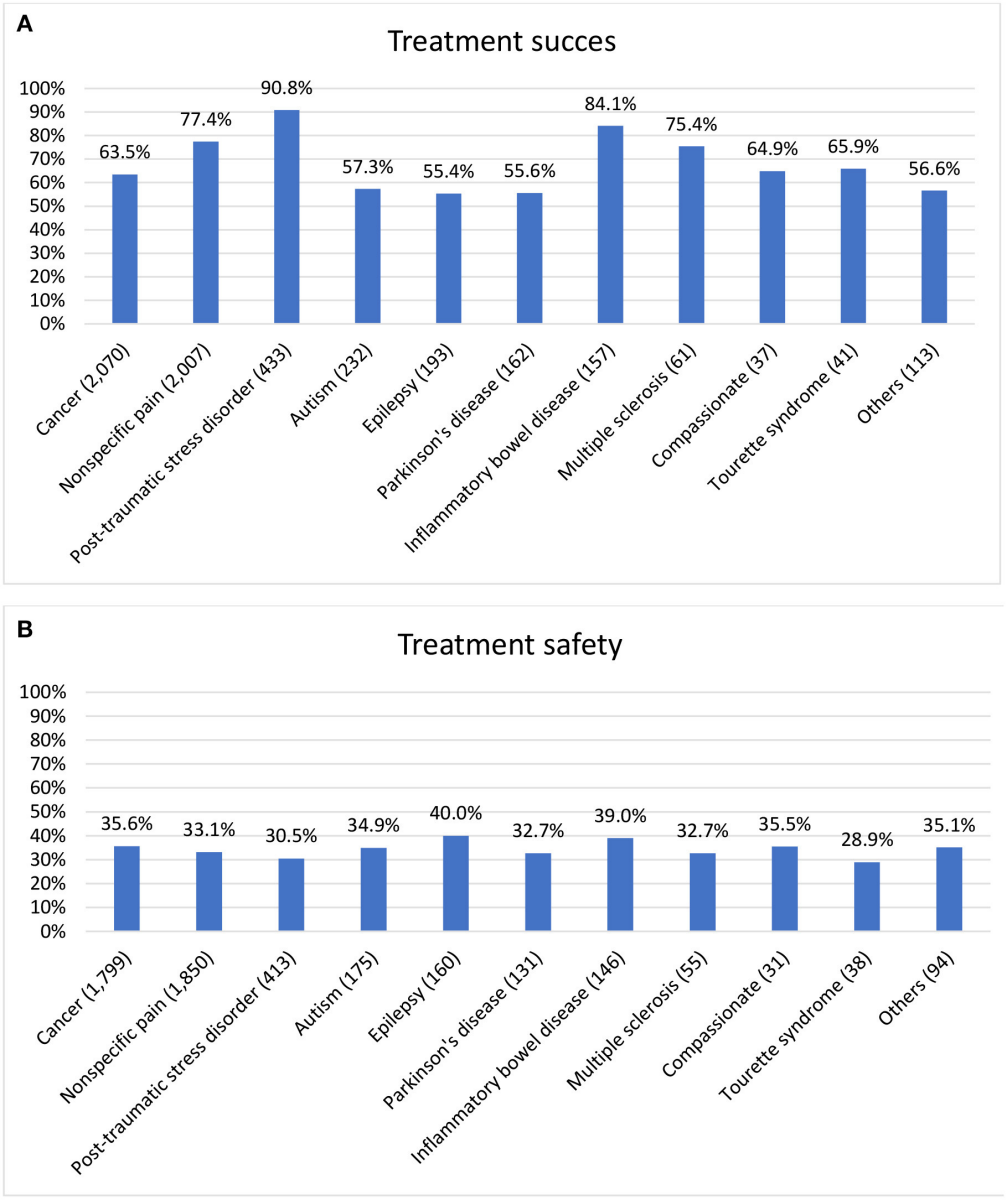
Safety and effectiveness rates by indications. **(A)** Treatment success in 4,345 patients who responded to the 6-month follow-up questionnaire (and to the general effect question) and in 1,160 patients who discontinued treatment, by the primary indication for the cannabis treatment. Treatment success was defined as at least a moderate or significant improvement in the patient's condition and none of the following: cessation of treatment or serious side-effects defined as 9–10 out of 10 on the severity scale. **(B)** Treatment safety—presence of any side-effect in 4,891 active and inactive patients who responded to the side-effect questions at the 6-months follow-up questionnaire, by the primary indication for the cannabis treatment.

Figure 4A presents rates of the primary outcome of treatment effectiveness and safety at 6 months, stratified by the primary indication for use, ranging between 55.4% for epilepsy to 90.8% for PTSD. Figure 4B presents the proportion of patients experiencing any side-effect, and ranges between 28.9% for Tourette syndrome to 40.0% in patients with epilepsy.

In the analysis of the subgroup of 1,500 patients with only one chemovar used, we found significant differences in success rate. The two chemovars utilized by more than 50 patients that differ in the success rate were Alaska 91% chance of success vs. Avidekel with 66.4% (*p* < 0.001). However, these chemovars were utilized by patients with different medical conditions and therefore the direct comparison of the success rates is not fully informative. We have not found a difference of safety rates between the different chemovars (*p* > 0.05).

### Missing Data Analysis

We compared baseline characteristics between patients responding to the 6-month questionnaire (4,364) and active patients with an intake questionnaire, without the 6-month follow-up (911). Patients without follow-up data had less experience with cannabis prior to the treatment initiation, had lower rates of tobacco use, consumed fewer medications, and recorded lower rates of driving (Supplementary Table 5). Even imputing a worst-case scenario in which all patients unavailable for follow-up were categorized as “treatment failure,” most patients nonetheless achieved therapeutic success with a marked improvement in their condition (3,902 patients of 6,435, 60.6%). These patients were certainly not “lost to follow-up” because they were all active patients who came month after month to the medical dispensary to buy their monthly quota.

## Discussion

In this prospective study, we describe the characteristics and outcomes of approximately 10,000 patients treated with medical cannabis. Results showed high adherence, high safety with a low incidence of adverse events, and a high rate of effectiveness in the prescribed treatment, as well as a decrease in pain levels, improvement in QOL, and a reduction in the consumption of concomitant medications.

### Demographic Characteristics

The characteristics of medical cannabis users (age, severity of diseases, number of comorbidities, number of symptoms, number of medications, employment status, etc.) depend upon and are determined by the limitations and laws set by governmental and regulatory authorities. During the study period, Israeli national regulation of medical cannabis provided strict guidelines for the patients and their physicians on the use of the medication. The demographic and medical characteristics of our cohort differ from most reported populations. The Israeli medical cannabis patients are on average (55 years old) two and a half-decade older than patients in comparable reports (2, 8, 10, 12–16, 18–21), with a more balanced gender distribution (51.3% men compared to 60–80% in most studies) (2, 11, 13–19, 21–24). In the current cohort, the main indication for cannabis treatment was cancer (48.9%), while in other studies the main indications were pain (2, 10–12, 15, 18, 24, 25, 33–35), anxiety (13, 14, 36), and depression (19); cancer was diagnosed in only 7.4–11.4% of the patients (2, 10–12, 14, 15, 19, 24, 25).

Almost 20% of the study population died within the first 6 months of follow-up primarily due to malignancies (90.1%).

### Treatment Adherence

Adherence to cannabis treatment was 77.7%, similar to the treatment withdrawal of 23.8% that was found in a retrospective cohort study on medical cannabis patients with a mean age similar to the patients' ages in our study (33).

Treatment adherence in our cohort was favorably comparable to the expected adherence in patients taking chronic medications: in a systematic review of 76 studies, patients taking medication on a schedule similar to the cannabis treatment regimen of at least four times daily, demonstrated average adherence rates of 50% (range 31–71%) (37). Furthermore, in a study of long-term treatment with opioids, treatment was discontinued in 51% of the patients (38).

### Safety

The safety of cannabis treatment in this heterogeneous population of patients was found to be high, especially when compared with the safety of long-term opioid treatment. Side-effects of medical cannabis were infrequent, minor, and rarely the cause of discontinuation. The most common side-effect, dizziness, was reported by 8.2% of the active responders, while the usual prevalence of side-effects in patients on opioid therapy is substantially higher: more than 40% of the patients report dizziness, more than 35% report constipation, more than 30% report nausea, and more than 25% report fatigue (38). In addition, long-term opioid treatment is associated with sedation, cognitive impairment, depression, addiction (39), and subtle neuropsychological changes (40–42). These high-safety results are similar to a large, controlled study that prospectively measured the safety of a high-THC medical cannabis product in 215 patients treated in chronic pain clinics. The patients were compared with 216 patients in the clinics who did not use medical cannabis and were followed-up for 1 year. The adverse events in this study were modest, and no significant difference in the occurrence of serious adverse effects was found (43). These results may be attributed to the safety-focused approach implemented; a guided choice of chemovar and route of administration, a slow titration method, an initial follow-up after 1 month, and a follow-up after 6 months, could be the strategy that ensured that harms from medical cannabis were mitigated (44).

### Effectiveness

Although this study is observational and thus no causality can be established, the treatment seems effective in reducing pain, in increasing QOL, and in reducing concomitant medication consumption. In our cohort, the primary effectiveness outcome was achieved by more than 70% of the patients, while only 17.4% of the patients discontinued treatment. Although further head-to-head comparative study between opioids and cannabis for palliation is needed, our results demonstrate numerically comparable effectiveness in pain treatment (e.g., opioids treatment provides adequate relief for 70–90% of patients with cancer pain) (45). However, long-term opioid treatment in non-specific pain patients delivered good pain relief in only 51% of patients (37). Although Cochrane review of neuropathic pain treated with cannabis-based medicines against placebo, found a modest gain from 16 studies (*n* = 1,750) with 21 vs. 17% achieving a 50% reduction in pain; and 39 vs. 33% achieving a 30% reduction (46), a multiple-criteria decision analysis found that the benefit-safety profiles for cannabinoids were higher than for other commonly used medications for chronic neuropathic pain largely because they contribute more to QOL and have a more favorable side-effect profile (47). Furthermore, for patients with chronic pain, opioids may contribute to substantial functional impairment (48), so serious adverse effects of opioids may limit effectiveness in some patients, even if adequate analgesia is achieved (48). The lack of serious side-effects of broad-spectrum cannabis products together with the effectiveness albeit shown in the small studies makes cannabis products a possible alternative for the treatment chronic pain.

The fact that previous experience with cannabis was associated with a higher chance of treatment success, can suggest that the placebo effect contributed to the overall improvement, as an expectation of a positive influence may increase the magnitude of the placebo effect. Moreover, young patients (usually with fewer comorbidities) that drive, smoke cigarettes, and are employed seems more likely to experience and report improvement following treatment. It is also possible that patients who smoke cigarettes know how to perform the inhaling action and are more likely to benefit from the treatment.

The broad effect of medical cannabis treatment, which has a beneficial effect on a variety of symptoms, can potentially explain the reduction in drug consumption (especially of painkillers). Cannabis may be a viable alternative to opioids for those experiencing pain (49, 50).

Out of 1,160 patients responded to the intake questionnaire and discontinued treatment, 593 filled the follow-up questionnaire at 6 months. The most common reasons for discontinuing treatment were side-effects (25.0%), no therapeutic effect (24.6%), no longer a need for cannabis treatment (23.2%), or failed renewals of mandatory cannabis treatment licenses (6.8%). Furthermore, 44.3% of the patients who discontinued the treatment have reported at least moderate improvement in their symptoms following cannabis treatment. Even though all patients who discontinued treatment were classed as “treatment failures”, we have recorded high rates of treatment success.

Treatment with medical cannabis is complex for several reasons: (1) the multiplicity of potential treatment chemovars, (2) the multiplicity of consumption options, (3) and because each patient will receive a different therapeutic dose, patients need to “find” their therapeutic dose in a slow titration process that is dictated by the psychoactive effect and other treatment side-effects.

A significant percentage of patients expressed concerns about initiating cannabis treatment. In addition, in the short-term follow-up (after about a month of active treatment), a large group of patients needed additional consultation with an experienced cannabis clinic nurse in order to adjust the dosage or the treatment product, emphasizing the great importance of professional guidance and instruction during the first ~2 months of treatment. Without guidance, patients may take too high a dose, experience a side-effect, and abandon the treatment. In addition, without setting expectations regarding the patience required in the first weeks of treatment (until the body adapts to the product, and until reaching the therapeutic dose, especially with CBD products), the patient may conclude that, if after several attempts his condition does not improve, the treatment is unhelpful and so may eventually quit.

### Limitations

The present findings should be interpreted with caution for several reasons. This is an observational study and therefore no causality between cannabis therapy and improvement in patients' wellbeing can be established. Patients who seek cannabis therapy might not constitute a representative sample of the patients with a specific disease (self-selection bias). The QOL and symptoms changes were assessed by non-validated questionnaires (though, the assessment was based on frequently used qualitative scales). Unfortunately, we have no data on the blood pressure and blood sugar control in our study population. Therefore, we cannot speculate on the effect of the decrease of use of the blood pressure, diabetic, steroid medications observed in our population. We used data collected routinely as part of the treatment program; therefore, some information like monthly income and use of illicit substances was not available. Furthermore, 14.2% of the patients initiating the treatment refused to undergo medical assessment even at baseline; we therefore could not assess safety and effectiveness of the treatment in this specific group of patients. As we have measured the refill adherence rather than the consumption adherence, some inaccuracies can emerge from including the patients who have bought the medications but did not consume it. Lastly, while the response rate at 6 months in living patients was above 70%, because of our population's morbidity, many had died within first 6 months, making it impossible to assess the safety and effectiveness of cannabis treatment in that subset of patients.

## Conclusions

This is a large study describing certain characteristics of medical cannabis users in a tightly regulated environment. The treatment appears to be safe and efficacious. Establishing national and international clinical research programs to assess the true therapeutic effect of cannabis on various diseases is needed. To further elucidate the safety and effectiveness of medical cannabis therapy using objective measures, the next step requires the performance of high-quality double-blind placebo-controlled clinical trials.

## Data Availability Statement

The raw data supporting the conclusions of this article will be made available by the authors, without undue reservation.

## Ethics Statement

The studies involving human participants were reviewed and approved by the IRB at the Soroka University Medical Center, Beer-Sheva, Israel, study number: SCRC-0415-15. Written informed consent from the participants or their legal guardian/next of kin was not required to participate in this study in accordance with the national legislation and the institutional requirements.

## Author Contributions

LB-L and VN conceived the study, wrote the protocol, drafted the manuscript, and verified the underlying data. All authors acquired, analyzed, or interpreted the data. LB-L was the principal investigator and oversaw study design. All authors approved the final article.

## Funding

This study was funded by Tikun Olam - Cannbit Pharmaceuticals.

## Conflict of Interest

This study was supported by the funding from Tikun Olam - Cannbit Pharmaceuticals. The funder had the following involvement with the study: data collection. Lihi Bar-Lev Schleider is an employee of Tikun-Olam Cannbit Pharmaceuticals Ltd. The remaining authors declare that the research was conducted in the absence of any commercial or financial relationships that could be construed as a potential conflict of interest.

## Publisher's Note

All claims expressed in this article are solely those of the authors and do not necessarily represent those of their affiliated organizations, or those of the publisher, the editors and the reviewers. Any product that may be evaluated in this article, or claim that may be made by its manufacturer, is not guaranteed or endorsed by the publisher.

## Abbreviations

CBD: cannabidiol
IBD: inflammatory bowel disease
IMOH: Israeli Ministry of Health
IQR: interquartile range
MS: multiple sclerosis
PD: Parkinson's disease
PTSD: post-traumatic stress disorder
QOL: quality of life
THC: delta-9-tetrahydrocannabinol.
