# Supplementary material for: Adherence, Safety, and Effectiveness of Medical Cannabis and Epidemiological Characteristics of the Patient Population: A Prospective Study
Source: Front Med (Lausanne). 2022 Feb 9;9:827849. doi: 10.3389/fmed.2022.827849 (PMC8864967; doi:10.3389/fmed.2022.827849)
Supplement: Supplementary file 1 [file Data_Sheet_1.docx]

Supplementary Material

# Supplementary Tables

| **Disease** | **Total Responses, No. (%)** | **Median disease duration in years (IQR)** |
| --- | --- | --- |
| Cancer | 4,426 (52.1) | 1 (1 - 3) |
| Pain | 1,589 (18.7) | 5 (2 - 10) |
| Hypertension | 1,186 (14.0) | 10 (5 - 15) |
| Diabetes | 897 (10.6) | 10 (5 - 17) |
| Spinal Disk Herniation | 693 (8.2) | 6 (3 - 10) |
| Post-Traumatic Stress Disorder (PTSD) | 623 (7.3) | 7 (3 - 16) |
| Heart Disease | 515 (6.1) | 9 (4 - 15) |
| Fibromyalgia | 452 (5.3) | 5 (2 - 10) |
| Autism | 354 (4.2) | 9 (5 - 13) |
| Arthritis | 337 (4.0) | 10 (5 - 18) |
| Epilepsy | 298 (3.5) | 8 (4 - 15) |
| Parkinson's Disease and Parkinsonism | 265 (3.1) | 7 (4 - 13) |
| Excessive Fat in the Blood | 232 (2.7) | 10 (5 - 14) |
| Degenerative Disc Disease | 208 (2.4) | 9 (4 - 15) |
| Depression | 207 (2.4) | 6 (2 - 15) |
| Osteoporosis | 199 (2.3) | ( - ) |
| Accident | 186 (2.2) | 3 (2 - 9) |
| Crohn's Disease | 174 (2.0) | 9 (3 - 17) |
| Stroke | 134 (1.6) | 5 (2 - 10) |
| Asthma | 132 (1.6) | 20 (19 - 20) |
| Chronic Obstructive Pulmonary Disease (COPD) | 105 (1.2) | 6 (3 - 10) |
| Migraines | 101 (1.2) | 20 (10 - 20) |
| Multiple Sclerosis | 87 (1.0) | 8 (4 - 15) |
| Colitis | 81 (1.0) | 10 (3 - 16) |
| Thyroid Problems | 76 (0.9) | 10 (6 - 20) |
| Renal Failure | 67 (0.8) | 5 (2 - 10) |
| Anxiety Disorder | 66 (0.8) | 7 (3 - 13) |
| Glaucoma | 61 (0.7) | 10 (5 - 19) |
| Tourette syndrome | 54 (0.6) | 14 (8 - 20) |
| Cerebral Palsy (CP) | 53 (0.6) | ( - ) |
| Psoriasis | 45 (0.5) | 10 (3 - 20) |

**Supplementary Table 1.** Disease Prevalence and Duration. Each patient has one indication for the cannabis license, but usually more than one medical condition.

| Drug class | Total (8,560) | Cancer (4,205) | Non-specific Pain (2,515) | PTSD (551) | Autism (311) | Epilepsy (232) | PD (215) | IBD (190) | MS (79) | Compassionate (55) | Tourette syndrome (48) | Others (159) |
| --- | --- | --- | --- | --- | --- | --- | --- | --- | --- | --- | --- | --- |
| Opioids | 2,274 | 1,134 | 1,024 | 40 | 0 | 1 | 23 | 18 | 6 | 3 | 4 | 21 |
| Antidepressants | 2,094 | 795 | 796 | 262 | 29 | 12 | 100 | 14 | 25 | 8 | 11 | 42 |
| Antiepileptics | 1,834 | 547 | 744 | 154 | 43 | 217 | 37 | 6 | 19 | 14 | 8 | 45 |
| Drugs for peptic ulcer and gastroesophageal reflux disease (GERD) | 1,622 | 985 | 487 | 29 | 4 | 10 | 48 | 19 | 10 | 4 | 1 | 25 |
| Antithrombotic agents | 1,494 | 812 | 540 | 36 | 1 | 4 | 57 | 7 | 4 | 7 | 2 | 24 |
| Anxiolytics | 1,362 | 696 | 474 | 88 | 7 | 9 | 51 | 6 | 4 | 6 | 1 | 20 |
| Lipid modifying agents | 1,316 | 659 | 506 | 45 | 0 | 1 | 58 | 12 | 12 | 6 | 3 | 14 |
| Hypnotics and sedatives | 1,264 | 532 | 489 | 93 | 44 | 1 | 1 | 5 | 9 | 5 | 2 | 25 |
| Other analgesics and antipyretics | 1,051 | 602 | 373 | 18 | 1 | 3 | 24 | 14 | 7 | 1 | 2 | 6 |
| Ace-inhibitors | 721 | 353 | 287 | 13 | 0 | 2 | 42 | 5 | 4 | 3 | 0 | 12 |
| Blood glucose lowering agents, excluding insulin | 687 | 391 | 244 | 11 | 1 | 1 | 20 | 4 | 4 | 2 | 1 | 8 |
| Selective calcium channel blockers with mainly vascular effects | 682 | 379 | 252 | 8 | 0 | 0 | 26 | 1 | 4 | 0 | 2 | 10 |
| Corticosteroids for systemic use | 618 | 449 | 129 | 1 | 0 | 3 | 1 | 21 | 2 | 2 | 0 | 10 |
| Beta blocking agents | 564 | 294 | 209 | 11 | 4 | 2 | 32 | 2 | 1 | 1 | 0 | 8 |

**Supplementary Table 2.** Concomitant Medications Use at Intake by Medical Condition. Prevalence of main medications families that were consumed regularly during intake session for all the patients and per indication. PTSD/Post-traumatic stress disorder; PD/Parkinson's disease; IBD/Inflammatory bowel disease; MS/Multiple sclerosis.

|  | With cannabis previous experience (N=2,590) | Without cannabis previous experience (N=5,606) | P value |
| --- | --- | --- | --- |
| Mean age (SD) | 49.5 (17.6) | 56.7 (21.8) | <0.001 |
| Gender (male), No. (%) | 1,518 (58.6) | 2,697 (48.1) | <0.001 |
| Malignancy (Yes), No. (%) | 925 (35.7) | 3,072 (54.8) | <0.001 |
| Chronic pain (Yes), No. (%) | 2,121 (81.9) | 4,193 (74.8) | <0.001 |
| Cigarette smoking (Yes), No. (%) | 989 (38.2) | 1,054 (18.8) | <0.001 |
| Cannabis average monthly dose in gr (SD) | 26.6±14.3 | 28.7±15.6 | 0.23 |

**Supplementary Table 3.** Comparison between Patients with and without Cannabis Previous Experience.

| Symptoms  No. (%) | Change at six months | | | |
| --- | --- | --- | --- | --- |
|  | Symptom Disappeared | Symptom Improvement | No change | Symptom Deterioration |
| Sleep disturbances | 434 (12.1) | 2766 (77.0) | 361 (10.1) | 30 (0.8) |
| Weakness and fatigue | 226 (10.6) | 1272 (59.9) | 573 (27.0) | 53 (2.5) |
| Digestion problems | 324 (17.9) | 1008 (55.6) | 449 (24.8) | 33 (1.8) |
| Anxiety | 140 (7.7) | 1425 (78.3) | 231 (12.7) | (1.4) 25 |
| Restlessness | 133 (8.2) | 1318 (81.4) | 154 (9.5) | 15 (0.9) |
| Depression | 137 (7.2) | 1505 (79.5) | 230 (12.2) | 21 (1.1) |
| Lack of appetite | 337 (20.8) | 1079 (66.6) | 192 (11.9) | 12 (0.7) |
| Nausea | 389 (30.5) | 745 (58.4) | 128 (10.0) | 14 (1.1) |
| Movement limitation | 67 (4.9) | 580 (42.1) | 671 (48.7) | 60 (4.4) |
| Paresthesia | 161 (11.1) | 874 (60.2) | 386 (26.6) | 32 (2.2) |
| Spasticity | 152 (11.1) | 862 (62.8) | 337 (24.5) | 22 (1.6) |
| Dizziness | 189 (20.3) | 547 (58.6) | 180 (19.3) | 17 (1.8) |
| Agitation | 83 (8.0) | 841 (80.6) | 103 (9.9) | 16 (1.5) |
| Burning sensation | 128 (14.9) | 563 (65.5) | 162 (18.8) | 7 (0.8) |
| Dry mouth | 146 (20.2) | 231 (32.0) | 314 (43.4) | 32 (4.4) |
| Headache | 183 (22.5) | 506 (62.3) | 115 (14.2) | 8 (1.0) |
| Respiratory problems | 158 (25.3) | 271 (43.4) | 169 (27.1) | 26 (4.2) |
| Cognitive impairment | 61 (10.4) | 226 (38.6) | 277 (47.4) | 21 (3.6) |
| Tremor | 101 (18.4) | 314 (57.1) | 122 (22.2) | 13 (2.4) |
| Pruritus | 178 (31.6) | 272 (48.2) | 104 (18.4) | 10 (1.8) |
| Rage attacks | 68 (11.0) | 499 (80.6) | 47 (7.6) | 5 (0.8) |
| Visual impairment | 50 (12.1) | 59 (14.3) | 267 (64.5) | 38 (9.2) |

**Supplementary Table 4.** Changes in Symptoms at Six Months.

|  | With six-month follow-up (N=4,364) | Without six-month follow-up (N=911) | P value |
| --- | --- | --- | --- |
| Mean age (SD) | 50.8 (19.6) | 49.9 (21.9) | 0.38 |
| Gender (male), No. (%) | 2,301 (52.7) | 470 (51.5) | 0.53 |
| Working (Yes), No. (%) | 1,286 (29.4) | 252 (27.6) | 0.54 |
| Driving a car (Yes), No. (%) | 2,452 (56.1) | 471 (51.7) | <0.05 |
| Median number of hospitalization days in the past six months (IQR) | 0 (0-3) | 0 (0-4) | 0.07 |
| Median number of medications (IQR) | 4 (2-6) | 3 (2-6) | <0.05 |
| Previous experience with cannabis (Yes), No. (%) | 1,691 (38.7) | 276 (30.2) | <0.001 |
| Cigarette smoking (Yes), No. (%) | 1,371 (31.4) | 207 (22.7) | <0.001 |

**Supplementary Table 5.** Summary characteristics of patients with missing six-month follow-up data.
